# Recurrent Positivity of SARS-CoV-2 RNA in a Clinically Recovered COVID-19 Patient with End-stage Renal Disease: A Case Report

**DOI:** 10.31729/jnma.5249

**Published:** 2020-11-30

**Authors:** Richa Nepal, Kalyan Sapkota, Santosh Gurung, Pramod Paudel, Prateek Neupane, Kamlesh Kumar Sah

**Affiliations:** 1 Department of Internal Medicine, Bharatpur Hospital, Bharatpur, Province-3, Chitwan, Nepal

**Keywords:** *coronavirus disease*, *COVID-19*, *end-stage kidney disease*, *severe acute respiratory syndrome coronavirus-2*

## Abstract

Recurrent or persistent positivity of severe acute respiratory syndrome coronavirus-2 RNA in clinically recovered coronavirus disease patients has been reported worldwide. However, replication-competent live viruses were not recovered beyond two to three weeks from the onset of symptoms in mild to severe cases of coronavirus disease. End-stage renal disease is characterized by uremia induced immune dysfunction that increases the risk of infectious diseases including coronavirus disease. The clinical implications of recurrent or persistently positive severe acute respiratory syndrome coronavirus-2 RNA in immunocompromised patients are difficult to be generalized to findings as in immunocompetent patients. We report a case of end-stage renal disease with a recent history of recovered coronavirus disease pneumonia, who again presented with positive reverse transcriptase-polymerase chain reaction test for severe acute respiratory syndrome coronavirus-2 RNA.

## INTRODUCTION

The pandemic of Coronavirus Disease 2019 (COVID-19) has affected 213 countries with more than 18 million cases worldwide by 1st August, 2020.^1^ Persistent or recurrent positivity of severe acute respiratory syndrome coronavirus-2 (SARS-CoV-2) RNA in clinically recovered patients has been reported worldwide.^2,3^ Lan et al. reported positivity of SARS-CoV-2 RNA from the throat swab of COVID-19 recovered patients till the maximum of 50 days.^4^ Persistent or recurrent positivity in an immune-compromised patient poses a dilemma regarding the infectivity of the patient. We report a case of end-stage renal disease patient with a recurrent positive test for SARS-CoV-2 RNA with a history of apparent clinical recovery for recent COVID-19 pneumonia.

## CASE REPORT

A 31 years-old male returned from Saudi Arabia around 11 days before presentation, came to the emergency of Bharatpur Hospital on 2nd July 2020 with the chief complaints of cough, dyspnea and multiple episodes of vomiting of two days duration. The patient was working in Saudi Arabia for the last 5 years and was diagnosed to be a case of chronic kidney disease around two and a half months back. He was under thrice-weekly hemodialysis at a centre in Saudi Arabia. He gave the history of getting admitted in a hospital in Saudi Arabia on 27th May 2020 for complaints of fever, cough, expectoration, myalgia and headache of 4 days duration. His nasopharyngeal swab came positive for SARS-CoV-2 RNA on the next day of admission. Computed tomography (CT) of his chest was done which revealed bilateral multifocal areas of alveolar consolidation, interlobar septal thickening and ground-glass opacities, suggestive of bilateral patchy pneumonia with moderate left-sided and mild right-sided pleural effusion. He was treated in line of COVID-19 pneumonia with supportive measures, oxygen supplementation and hemodialysis. He gradually improved during the hospital stay and was discharged after two negative reverses transcriptase-polymerase chain reaction (RT-PCR) tests for SARS-CoV-2 on 5th and 10th June 2020. He was advised for thrice-weekly hemodialysis for his kidney disease.

The patient returned to Nepal on 23rd June 2020 and was advised to self-quarantine at his home in Hetauda. After eight days, he complained of cough with the passage of pink frothy sputum, exertional dyspnea and multiple episodes of vomiting, following which he was brought to Hetauda district hospital. He denied any history of fever, sore throat, ageusia, anosmia, rhinorrhea, headache or diarrhoea. Nasopharyngeal swab for SARS-CoV-2 was collected, as he was a foreign returnee with new-onset respiratory complaints with the possible need of hemodialysis. The report came out to be negative following which he was referred to Bharatpur Hospital for the need of hemodialysis.

The patient was evaluated at the emergency of Bharatpur Hospital where his blood pressure was 180/130 mmHg, respiratory rate was 28/min and oxygen saturation was 93% in room air. On examination, there was bilateral pitting pedal oedema, and chest auscultation revealed decreased air entry in the left infra-axillary area with bilateral infrascapular inspiratory crepitations. Table 1 shows the baseline investigations of the patient (Table 1).

**Table 1:** Baseline investigations of the patient on the day of the presentation.

Hemoglobin	8.2gm/dL
WBC count (N/L/E/M/B)	7450/cumm (71/15/5/9/0)
Platelet count	140000/cumm
Urea	330mg/dL
Random blood sugar	106.5mg/dL
Creatinine	24.4mg/dL
Sodium	129meq/dL
Potassium	5.3mg/dL
Total bilirubin	0.7mg/dL
Direct bilirubin	0.2mg/dL
Alanine transferase (ALT)	14.4IU/L
Aspartate transferase (AST)	10.4IU/L
Alkaline phosphatase	71.0U/L
ECG	Normal sinus rhythm
USG abdomen and pelvis	Bilateral renal disease with loss of cortico-medullary differentiation

**Figure 1:**
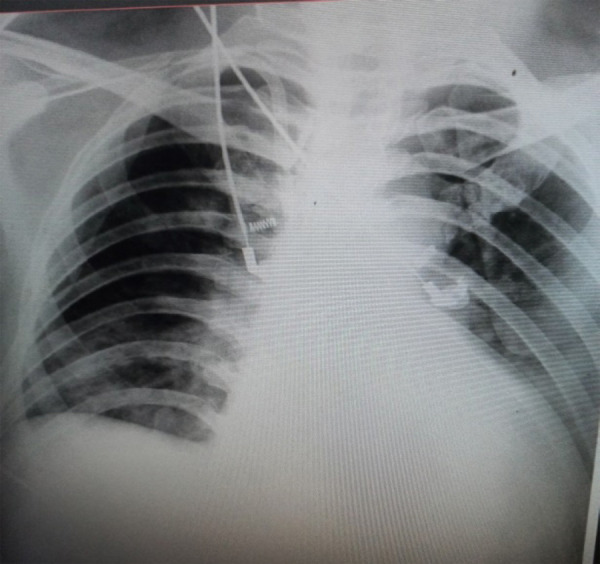
Chest X-Ray (Antero-posterior view) taken in the intensive care unit on the second day of admission.

Chest X-ray revealed homogenous opacity involving bilateral lower zones with blunting of left costophrenic angle and cephalization of pulmonary vessels (Figure 1).

The provisional diagnosis of the patient was made as acute pulmonary oedema secondary to volume overload with underlying end-stage renal failure. Two consecutive RT-PCR tests of the patient, which was done in Saudi Arabia, was negative for SARS-CoV-2; thus the patient was considered clinically recovered from his recent COVID-19 infection. However, a nasopharyngeal swab of the patient for RT-PCR of SARS-CoV-2 was collected because of underlying immunocompromised status and his new respiratory complaints. He was shifted to a separate intensive care unit allocated for the suspected COVID-19 cases, from where urgent hemodialysis was planned. The symptoms of patient drastically improved after hemodialysis.

**Figure 2:**
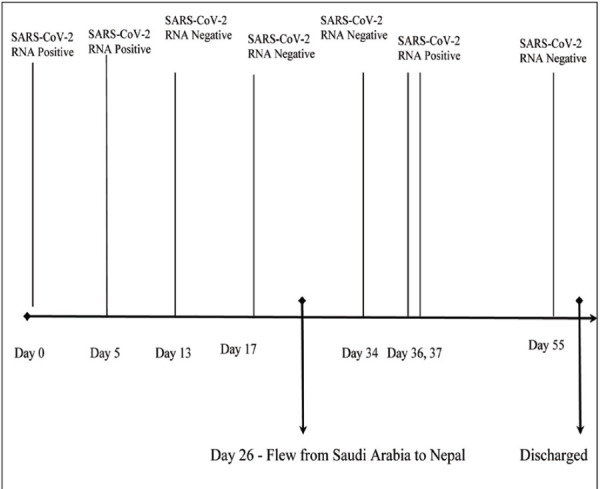
Timeline showing the profile of SARS-CoV-2 RNA in the ESRD patient.

On 4th July 2020, the RT-PCR for SARS-CoV-2 of the patient, which was collected during the time of admission, came positive. To re-confirm, RT-PCR was repeated the next day, which also came positive. Because of the immunocompromised status of the patient due to underlying end-stage renal disease, reinfection or reactivation could not be ruled out. Thus, contact tracing was done and all the health care workers, who were close contacts of the patient in the last 48 hours, were quarantined. Necessary decontamination of the intensive care unit and hemodialysis unit was done. The patient was shifted to COVID unit, where new setup for hemodialysis was made and maintenance hemodialysis was continued with necessary transmission-based precautions. The patient developed no fever during the hospital stay and had no respiratory complaints following hemodialysis. The course of stay in hospital was uneventful. None of his close contacts, which included both the healthcare workers and his family members, were positive for SARS-CoV-2 RNA. Finally, his RT-PCR for SARS-CoV-2 came negative on 23rd July 2020. He underwent six sessions of hemodialysis during the hospital stay and was finally discharged after twenty-five days of admission. Figure 2 shows the timeline of SARS-CoV-2 RNA in the ESRD patient.

## DISCUSSION

According to the latest Centers for Disease Control and Prevention (CDC) guidelines, the test-based strategy is not recommended to determine when to discontinue transmission-based precautions in a COVID-19 patient. Apart for the criteria of symptomatic improvement, CDC recommends transmission-based precautions can be discontinued after 10 days from the day of onset of symptoms in patients who have mild to moderate disease, whereas transmission-based precautions can be discontinued after 20 days from the day of onset of symptoms in patients with severe disease or in those who are severely immunocompromised.^5^

The test-based strategy is only implemented for severely immunocompromised patients if there are concerns for infectivity beyond 20 days from the onset of symptoms. However, the criteria for severe immunocompromise have not been clearly defined. Ultimately, the degree of immunocompromise for the patient is determined by the treating doctor.^5^ End-stage renal disease is characterized by uremia induced immune dysfunction and immunodepression, that contributes to the high prevalence of infectious diseases in these patients.^6^ Infection is the second most common cause of mortality among ESRD patients after cardiovascular diseases.^7^ Persistence or recurrence of SARS-CoV-2 RNA in immunocompromised patients is difficult to be dismissed as inactive viral particles as in immunocompetent patients. A study by Sun et al. reported the estimated time until loss of viral RNA from nasopharyngeal samples to be 45.6 days for mild cases and 48.9 days for severe cases.^8^ However, infectious viral particles were not demonstrated beyond eight days from the symptom onset in mild COVID-19 cases and beyond 20 days from the symptom onset in severe COVID-19 cases.^9^ Despite these findings, it is necessary to consider the fact that these are not the studies done among patients with known immune dysfunction. Evidences are not sufficient to generalize the results to high-risk patients like that of end-stage renal disease.

Data are limited regarding the longevity of antibody response to SARS-CoV-2 following the clinical recovery of primary infection, but antibody response to other homologous coronaviruses is known to wane over time, and reinfection has been documented as early as 80 days.^10^ Reinfection of COVID-19 could not be ruled out in areas where the risk of infection is sustained. CDC strongly recommends retesting for COVID-19 if symptoms reappear after three months of the onset of symptoms of primary COVID-19 infection, whereas retesting should be considered after ruling out alternative diagnoses if symptoms reappear within three months of the onset of symptoms of the recent COVID-19 infection.^11^ However, the contagiousness of such re-infected patients is a matter of debate. Until further evidence exists, such patients are recommended to be isolated till they again meet the criteria for discontinuation of transmission-based precautions.^11^

The recurrence of SARS-CoV-2 RNA in our patient, who had clinically recovered from COVID-19 pneumonia about three weeks before presentation, could have been due to reactivation of the virus, reinfection with the virus, false-negative at the time of discharge or intermittent positive result due to persistent sub-genomic inactive viral particles. The patient did not develop clinical symptoms suggestive of COVID-19 pneumonia during the hospital stay. However, transmission-based precautions could not be de-escalated due to inability to rule out live viral shedding in an immunocompromised patient. The inability to de-escalate infection prevention measures prolonged the hospital stay of the patient and added to the economic burden of the resource-limited setup.

Ruling out contagiousness of a patient in special circumstances like these, warrants further tests like isolation of live virus through viral culture or cycle threshold (Ct) value of reverse transcriptase-polymerase chain reaction. Evidences suggest that live viruses were not isolated from samples when the cycle threshold (Ct) values during RT-PCR were above 30-34 and it could be used as a guide to discontinue transmission-based precautions.^12^ Vankampen et al. recommended the combined use of quantitative viral RNA load assays and serological tests to decide on de-escalation of infection control measures in COVID-19 patients. Infectious viral shedding dropped to undetectable levels below a threshold of viral RNA load and with the appearance of neutralizing antibodies.^9^ Thus, it is high time that quantitative testing and quantitative reporting system must be developed rather than just qualitative assays for diagnosis of COVID-19.

This was the first COVID-19 case that underwent hemodialysis at Bharatpur Hospital. In a cohort of 5700 COVID-19 patients admitted to different hospitals in New York, 3.4% were ESRD patients.^13^ The pandemic of COVID-19 poses an additional threat to ESRD patients who are dialysis-dependent for survival and are prone to develop severe disease. Hemodialysis is a lifesaving procedure that needs to be acknowledged as a part of critical care for these severely ill COVID-19 patients with renal impairment. With the rising number of COVID-19 cases in Nepal, it is high time that we prepare for the possible surge of COVID-19 infection among ESRD patients and make necessary logistic arrangements to provide hemodialysis services to them.
